# Assessment of endometrial and ovarian characteristics using three dimensional power Doppler ultrasound to predict response in frozen embryo transfer cycles

**DOI:** 10.1186/1477-7827-7-151

**Published:** 2009-12-25

**Authors:** Tamara Žáčková, Ilkka Y Järvelä, Juha S Tapanainen, Jaroslav Feyereisl

**Affiliations:** 1 Department of Obstetrics and Gynecology, Oulu University Hospital, Oulu, Finland; 2 Institute for the Care of Mother and Child (UPMD), Department of IVF, Charles University, Prague, Czech Republic

## Abstract

**Objective:**

To evaluate whether endometrial or ovarian parameters as measured using 3D power Doppler ultrasound would predict the outcome in frozen embryo transfer (FET) cycles.

**Methods:**

Thirty women with no known gynecological pathology undergoing FET were recruited. The FET was carried out in the natural menstrual cycle 3-4 days after the first positive LH test result. Blood samples for hormonal analysis were collected, and three-dimensional (3D) ultrasonographic examination was performed on the day of the FET and repeated with analysis of the total hCG one week later.

**Results:**

The demographic, clinical, and embryological characteristics were similar between the pregnant (15/30) and nonpregnant groups (15/30). There were no differences between the groups in endometrial/subendometrial thickness, volume, or vascularization index (VI). The endometrial triple-line pattern was more often present in the pregnant group on the day of the FET (93.3% vs. 40.0%, 95% CI 25.5-81.2%). No differences in the ovaries were observed on the day of the FET. At the second visit, the triple-line pattern was still more often present in those patients who had conceived (91.7% vs. 42.9%, 95% CI 18.5-79.1%), and their corpus luteum was more active as judged by the rise in 17-hydroxyprogesterone and estradiol levels. No differences were observed in the dominant ovarian vasculature.

**Conclusions:**

According to our results, measurement of power Doppler indices using 3D ultrasound on the day of the FET does not provide any additional information concerning the outcome of the cycle. The existence of the triple-line pattern on the day of the FET seems to be a prognostic sign of a prosperous outcome after FET. The dominant ovary in the pregnant group seems to be already activated one week after the FET.

## Background

Because of wider use of elective single embryo transfer, frozen-thawed embryo transfers (FETs) have also become more common. According to European data for artificial reproductive treatment (ART) [1], 64,147 FETs were performed during 2004 in Europe, and an approximate clinical pregnancy rate was 19.1%. The highest rates of single embryo transfers (SETs) from all transfers were found in Sweden (67%), Belgium (49%), and Finland (47%) [1]. During 2005 and 2006 in Finland, the number of FETs increased by 7% while the number of IVF and ICSI cycles remained unchanged. In 52.7% of all the FET cycles in Finland, only one embryo was transferred [2]. The overall live birth rate/cycle after FET was 16.6%, and after single embryo transfer the rate was 14.2% and 19.3% after double embryo transfer [2]. In 2006, every fourth child born after ART originated from an FET cycle [2], emphasizing the role of FET cycles in Finnish ART practice.

Since less than 20% of FET cycles result in pregnancy and live birth, it is important to evaluate factors that may affect the treatment result. It seems that failure of implantation remains the main reason why most treatments fail [3]. The failure of implantation may be caused by the low quality of the embryo(s) transferred or by a nonreceptive uterine endometrium. If the nonreceptive endometrium could be detected before the transfer of the embryo, the cycle could be abandoned, and the embryo(s) could be transferred during a cycle with more favorable circumstances.

The ability to identify a receptive uterus prospectively by a noninvasive method would have an invaluable clinical impact on treatment efficiency and success rates. Ultrasonographic examination offers us a noninvasive method to evaluate the uterine environment before embryo transfer. Several sonographic parameters have been proposed for assessing endometrial receptivity, including endometrial thickness, endometrial pattern, endometrial volume, and endometrial and subendometrial blood flow [4-7]. The aim of this prospective study was to evaluate whether any of the endometrial or ovarian parameters measured using 3D power Doppler ultrasound before the transfer of frozen and thawed embryos have an impact on the outcome of the cycle. The ultrasonographic parameters of the endometrium and ovaries were correlated with the systemic steroid concentrations. In addition, we wanted to evaluate whether these parameters differed one week after embryo transfer--at the time of presumed implantation--between those who conceived and those who did not.

## Methods

We recruited 30 women undergoing frozen-thawed embryo transfer (FET) during a natural menstrual cycle between May 2004 and February 2008 in the Assisted Reproduction Unit of the Department of Obstetrics and Gynecology, Oulu University Hospital, Finland. The study was approved by the Ethics Committee of Oulu University Hospital, Oulu, Finland. Informed written consent was obtained from all participants, after the nature of the procedures had been fully explained. Inclusion criteria were women with no known gynecological pathology (e.g., known endometriosis, fibroids, any previous operation to gynecological organs). For the study, the patients were examined on the day of the FET and 5-9 days later. Blood samples for hormonal assays were collected, and three-dimensional (3D) ultrasonographic examination was performed during both visits.

Indication for previous IVF or ICSI treatment included male, tubal, anovulation, endometriosis, mixed, and unexplained factors. ICSI was performed for 16 couples with severe semen abnormalities (Table 1). The details of the long protocol for ovarian stimulation have been described previously [5]. During the IVF/ICSI treatment, the excess embryos were frozen after culture for two days using a slow-freezing protocol with 1,2-propanediol as cryoprotectant [8].

**Table 1:** Demographic, clinical, and embryological characteristics for the pregnant and nonpregnant groups

	Pregnant (n = 15)	Nonpregnant (n = 15)	P- value
**Age (years)**	31,3 ± 1,1	31,5 ± 1,1	0,905
**BMI (kg/m2)**	22,9 ± 1,1	23,0 ± 0,8	0,941
**Partus**	0,2 ± 0,1	0,5 ± 0,3	0.372
**Grav**	0,5 ± 0,2	0,5 ± 0,3	0.843
**Causes of infertility; n (%)**			
**Male**	8(53,3)	10 (66,7)	0.738
**Tubal**	1(6,7)	1 (6,7)	
**Anovulation**	1(6,7)	0 (0)	
**Endometriosis**	2 (13,3)	1 (6,7)	
**Mixed**	0 (0)	1 (6,7)	
**Unexplained**	3 (20,0)	2 (13,3)	
**Number of previous IVF**	1,5 ± 0,3	1,7 ± 03	0.853
**Number previous FET**	0,9 ± 0,3	0,9 ± 0,2	0.533
**Number of transfered embryos; n (%)**	1,5 ± 0,1	1,5 ± 0,1	1.000
**Insemination Method; n (%)**			0.075
**IVF**	10 (66,7)	4(26,7)	
**ICSI**	5 (33,3)	11(73,3)	
**LH test positive, cycle day**	14,7 ± 0,5	13,8 ± 0,7	0,246
**Embryotransfer, cycle day**	18,0 ± 0,6	17,2 ± 0,6	0,368

Values are mean ± SEM or n (%).

No statistically significant differences were observed between the pregnant and nonpregnant groups.

In the FET cycles, ovulation occurred spontaneously, without any kind of hormonal treatment. According to the FET protocol used in our clinic, the transvaginal ultrasonographic examination was carried out during cycle days 10-12, and the side and size of the dominant follicle and endometrial thickness were recorded. Endometrial thickness was measured as the thickest part in the longitudinal section, including both endometrial layers. After the ultrasonographic examination, the women started luteinizing hormone (LH) tests on urine samples every morning to confirm ovulation (home test kit, Clearplan; Unipath, Bedford, UK). The embryo transfer was carried out 3-4 days after the first positive LH test result. A maximum of two embryos were transferred. The thawed embryos were graded using the same criteria as in the fresh cycles [9]. A top-quality embryo was defined as having four blastomeres on day 2 or ≥ 8 blastomeres on day 3, less than 20% fragmentation, and no multinuclear blastomeres. After the transfer, the patients started to use 200 mg of progesterone twice a day either per os or intravaginally, depending on their preference. In case of pregnancy, the progesterone treatment was continued up to pregnancy week 10.

The 3D transvaginal ultrasound measurements (Voluson Expert 730, Kretz, Zipf, Austria) were performed on the day of the FET and repeated about one week later, at the time of the expected implantation. The results of the ultrasound examination did not affect subsequent clinical management procedures. The 3D ultrasound technique enabled the determination of endometrial, subendometrial, and ovarian volume, including possible changes in the vascular network. Identical preinstalled instrument settings (frequency, mid; dynamic set, 2; balance, G>170; smooth, 5/5; ensemble, 16; line density, 7; power Doppler map, 5; and the setting conditions for the power Doppler mode were gain, -5.6; quality, normal; wall motion filter, low 1; peak repetitive frequency PRF, 0.9 kHz) were applied for all patients. At the second visit, the power Doppler mode was not used to examine the uterus.

During the ultrasound examination, the uterus was first visualized in two-dimensional (2D) B-mode after the patient had emptied her bladder. The power Doppler mode was switched on, and the power Doppler box was positioned to cover the whole uterus. The 3D facility was engaged by switching to 'volume mode.' The volume sector angle was preset to 80°, and the fast volume acquisition (low-resolution) setting was selected to avoid artifacts. Thereafter, the ovaries were examined similarly. The ultrasonographic volume data were saved on the hard drive and analyzed later using the built-in virtual organ computer-aided analysis imaging program (VOCAL, GE Healthcare, Zipf, Austria) for 3D power Doppler histogram analysis. The manual mode of the VOCAL Contour Editor was used to cover the whole 3D volume of the region of interest (ROI), with 15° rotation steps. Hence, 12 contour planes were analyzed for each ROI to cover 360°. After obtaining the total volume of the ROI, the program calculated the ratio of color voxels to all the voxels; this ratio (%) was expressed as the vascularization index (VI). The vascularized volume (unit mL) in the endometrium or in the ovary was calculated by multiplying the total volume of the ROI by its VI. The VOCAL program automatically calculated the index for mean grayness (MG) in the ROI, which presents the average grayness in the gray-scale voxels [10].

The endometrial thickness, including both endometrial layers, was measured at its thickest in the longitudinal section. The endometrial pattern was assessed from the same section and described as triple-line or homogeneous. The echogenity of the endometrium in comparison with the subendometrium was evaluated with two different methods. First, a single observer (T.Z.) evaluated the endometrial echogenity subjectively and categorized it as either hyperechoic, homoechoic, or hypoechoic in comparison with the myometrial echogenity. In the second method, we used the MG values supplied by the VOCAL. The MG_endom _is the mean gray value in the endometrium, and the MG_subendom _is the mean gray value in the subendometrium. The subendometrial volume was calculated after we had defined the subendometrial region as the region 5 mm beneath the myometrial-endometrial junction. The reproducibility of the volume and color index measurements using transvaginal 3D power Doppler ultrasound has been determined [11]. The reproducibility of volume and color index measurements using transvaginal 3D power Doppler ultrasound has been determined by Järvelä et al 2003 [11]. The intraobserver correlation coefficient for volume measurements was 1.0 and for VI it was 0.898[11].

### Assays

The blood samples for analysis of serum progesterone (P), 17-hydroxyprogesterone (17-OHP), and estradiol (E_2_) were collected on the day of the FET and repeated about one week later with the analysis of total human chorionic gonadotrophin (hCG). Clinical pregnancy was confirmed by ultrasonography after a positive urinary pregnancy test and visible heartbeat at pregnancy week 7.

Serum concentrations of P and total hCG were measured by an automated chemiluminescence system (Advia Centaur, Bayer Corporation, NY, USA), 17OHP by radioimmunoassay (Diagnostic Products Corporation, LA, USA), and E_2_by radioimmunoassay (Orion Diagnostica, Oulunsalo, Finland), following the instructions provided by the manufacturers.

The intra-assay and the interassay coefficients of variation were 3.7% and 5.4% for P, 5.0% and 5.4% for 17-OHP, 2.0% and 3.5% for hCG, and 5.7% and 6.4% for E2. External quality control of the hormone assays was organized by national (Labquality Ltd., Helsinki, Finland) and international (Bio-Rad Laboratories EQAS, Irvine, CA) companies.

### Statistics

Statistical analysis was performed using the Statistical Package for the Social Sciences (Release 16.0, SPSS Inc., Chicago, IL, USA). Departure from a normal distribution was assessed using the Kolmogorov-Smirnov test. For paired comparisons, the paired *t*-test for normal data or Wilcoxon's test for skewed data was used. Correlation was estimated using Pearson's correlation coefficient. Proportions were compared using the chi-square test. A *p*-value (two-tailed) of < 0.05 was considered significant.

## Results

A total of 30 women undergoing FET were recruited for the study. Four patients missed the second set of measurements. The pregnancy rate was 50% (15/30). For statistical analysis, the women were divided into two groups according to whether or not they had conceived. These two groups did not differ from each other in demographic, clinical, or embryological characteristics (Table 1).

There were no differences between the pregnant and nonpregnant groups in the 3D-ultrasound characteristics of the ovaries and serum steroid levels on the day of the FET (Table 2). The endometrial triple-line pattern was more often present in the pregnant group on the day of the FET (93.3% *vs*. 40.0%, *p*-value of chi-square test 0.005). In the group of triple-line patients, the pregnancy rate was 70% (14/20), while for the group with the homoechogenic endometrium, the pregnancy rate was only 10% (1/10). The patients were further divided into two groups according to an endometrial cutoff volume of 2 ml. In the group of patients whose endometrial volume was more than 2 ml, the pregnancy rate was 55% (12/22), whereas in the group whose volume was less than 2 ml, the rate was 38% (3/8) (NS).

**Table 2:** Comparison of ultrasonographic and hormonal parameters on the day of FET between the pregnant and nonpregnant groups.

	Pregnant (n = 15)	Nonpregnant (n = 15)	P- value
**Endometrial thickness (mm)**	11,1 ± 0,3	11,2 ± 0,7	0,882
**Endometrial Volume (ml)**	3,7 ± 0,4	3,4 ± 0,7	0,669
**Endometrial pattern triple line** **(n, %)**			**0.005**
**Homoechogenic**	1 (6,7)	9 (60,0)	
**Tripple line**	14 (93,3)	6 (40,0)	
**Endometrial vascularization index (VI) %**	0,9 ± 0,3	1,1 ± 0,4	0,661
Endometrial vascularization** (ml)	0,04 ± 0,02	0,04 ± 0,02	0,887
**Subendometrial Volume (ml)**	11,2 ± 0,9	11,3 ± 1,2	0.996
**Subendometrial vascularization index(VI) %**	4,1 ± 1,7	1,2 ± 0,4	0,110
Subendometrial vascularization** (ml)	0,5 ± 0,1	0,2 ± 0,1	0,075
Endometrial echogenity* Subjective evaluation (n,%)			
**Hyperechogenic**	15 (100)	15 (100)	
**Hypoechogenic**	0 (0)	0 (0)	
**MG endsubend**	1,24 ± 0.05	1,25 ± 0,06	0,917
**MG subend**	29,25 ± 1,60	26,95 ± 2,56	0,453
**Dominant -ovary Volume (ml)**	17,0 ± 2,9	15,1 ± 1,8	0,562
**Dominant ovary vascularization index (VI) %**	9,2 ± 1,5	7,6 ± 1,1	0,382
Dominant ovary vascularization** (ml)	1,4 ± 0,2	1,0 ± 0,1	0.134
**Nondominant ovary Volume (ml)**	9,0 ± 1,1	7,8 ± 0,7	0,376
**Nondominant-ovary vascularization index(VI) %**	3,7 ± 0,7	5,2 ± 1,2	0,287
Nondominant ovary vascularization** (ml)	0,4 ± 0,1	0,4 ± 0,1	0,725
**Progesterone on the day of FET (nmol/l)**	25,4 ± 3,2	45,9 ± 12,7	0,130
**17-OHP on the day of FET (nmol/l)**	8,6 ± 0,4	9,6 ± 0,8	0,263
**Estradiol on the day of FET (nmol/l)**	0,31 ± 0,02	0,33 ± 0,03	0,695

Values are mean ± SEM or n (%).

*The echogenity of the endometrium in comparison with subendometrium was evaluated by two different methods, which were subjective analysis and mean gray (MG) -value supplied by VOCAL.

MG_endom_, mean gray value in the endometrium; MG_subendom _mean gray value in the subendometrium. **Vascularization, volume multiplied with VI.

The endometrial echogenity at the time of the FET was evaluated using two methods (Table 2). First, a single observer evaluated subjectively the endometrial echogenity and categorized it as either hyperechoic, homoechoic, or hypoechoic in comparison with the subendometrial (myometrial) echogenity. In the second method, we used the mean gray (MG) values that were calculated by VOCAL for both the endometrial and subendometrial volumes. The MG_endom _is the mean gray value in the endometrium, and the MG_subendom _is the mean gray value in the subendometrium. The difference (MG_endom _- MG_subendom_) ranged from 0.37 to 15.87 (mean 6.36 ± 0.91) and was above 0 in all cases. The subjective evaluation resulted exactly in the same finding as the method using VOCAL, i.e., the endometrium was more echogenic in the ultrasound than the subendometrium.

The characteristics and outcomes of the treatment cycles about one week after the FET, at the time of expected implantation, are given in Table 3. Four patients (three pregnant and one nonpregnant) missed the second set of measurements for personal reasons. During the second examination, the power Doppler mode was not used to investigate the uterus, and therefore, the data concerning the endometrium/subendometrium does not include any power Doppler information. The triple-line pattern was still more often present in those patients who conceived (91.7% *vs*. 42.9%, 95% CI for the difference between proportions 18.5%-79.1%). The endometrium was also significantly thicker in the pregnant group at this stage. No differences were observed in the dominant or in the nondominant ovarian vasculature parameters between the pregnant and nonpregnant groups. The pregnant group had significantly higher 17-OHP (p 0,009) and estradiol levels (p 0,039) than the nonpregnant group one week after the FET. Out of those 12 patients who finally conceived and were examined twice, 6 had a positive hCG pregnancy test at the 2^nd ^measurement, which took place 7.2 ± 0.2 days after the FET. Another 6 patients were tested negative at their second visit (5.3 ± 0.4 days after the FET), but they showed a positive result of hCG pregnancy test later.

**Table 3:** Comparison of ultrasonographic and hormonal parameters one week after FET between the pregnant and nonpregnant groups.

	Pregnant (n = 12)	Nonpregnant (n = 14)	P- value
**Days after FET**	6,3 ± 0,4	5,9 ± 0,4	0,539
**Endometrial thickness (mm)**	12,5 ± 0,6	10,6 ± 0,6	**0,049**
**Endometrial Volume (ml)**	4,3 ± 0,7	4,1 ± 0,9	0,925
**Endometrial pattern triple line (n, %)**			**0.019**
**Homoechogenic**	1(8,3)	8(57,1)	
**Tripple line**	11(91,7)	6(42,9)	
**Dominant ovary Volume (ml)**	15,3 ± 1,5	12,5 ± 1,8	0,255
**Dominant ovary vascularization index (VI) %**	10,5 ± 1,8	8,6 ± 1,63	0,446
Dominant ovary vascularization **(ml)	1,4 ± 0,2	0,9 ± 0,1	0.084
**Nondominant ovary Volume (ml)**	9,4 ± 1,5	8,2 ± 0,8	0,454
**Nondominant ovary vascularization index(VI) %**	3,3 ± 0,7	6,1 ± 1,3	0,072
Nondominant ovary vascularization ** (ml)	0,3 ± 0,1	0.5 ± 0,1	0,118
**Progesterone one week after FET(nmol/l)**	174,5 ± 68,0	92,3 ± 18,3	0,224
**17-OHP one week after FET(nmol/l)**	15,1 ± 2,1	8,3 ± 1,3	**0,009**
**Estradiol one week after FET(nmol/l)**	0,40 ± 0,06	0,25 ± 0,04	**0,039**

Four patients missed the second set of measurements. The power Doppler mode was not applied at any time during the visit to investigate the pregnancy itself (the fetus or the trophoblastic tissue). Therefore, data concerning the fetus, gestational sac, or placenta do not include any power Doppler information.

**Vascularization, volume multiplied with VI. Values are mean ± SEM or n (%). 17-OHP = 17 hydroxyprogesterone

The change of ultrasonographic and serum hormone levels between the first and second examination was also compared (Table 4). In the 12 pregnant patients, there was an increase in P- and 17-OHP levels and in endometrial thickness. In the 6 patients with a positive pregnancy test at the second visit, a similar rise in P- and 17-OHP levels and in endometrial thickness was also observed (Table 4). Another 6 patients were tested negative at their second visit, but they showed a positive result of hCG pregnancy test later, only their P levels rose between the day of FET and the day of the second visit (Table 4).

**Table 4:** Subanalysis of serum hormone concentrations and endometrial thickness one week after FET.

	Pregnant (n = 12)	P	Pregnant, hCG positive (n = 6)	P	Pregnant, hCG negative (n = 6)	P	Nonpregnant (n = 14)	P
**P1**	25,1 ± 3,3	**0.002**	24,2 ± 6,5	**0.005**	26,0 ± 2,4	**0.028**	48,7 ± 13,3	**0.041**
**P2**	174,5 ± 8,0		121,2 ± 23,9		227,8 ± 136,5		92,3 ± 18,3	
**17-OHP1**	9,0 ± 0,5	**0.015**	9,2 ± 0,8	**0.046**	8,8 ± 10,5	0.249	9,9 ± 0,8	0.300
**17-OHP2**	15,1 ± 2,1		16,9 ± 2,6		13,2 ± 3,3		8,3 ± 1,3	
**Endometrial thickness 1 (mm)**	11,2 ± 0,4	**0.006**	**11,4 ± 0,8**	**0.028**	10,9 ± 0,2	0.075	11,2 ± 0,8	0.132
**Endometrial thickness 2 (mm)**	12,5 ± 0,6		12,3 ± 1,0		12,7 ± 0,9		10,5 ± 0,6	

P, progesterone; 17-OHP, 17-hydroxyprogesterone; Values are mean ± SEM or n (%).

Index 1 = measurements on the day of FET, Index 2 = one week later, at the time of second visit.

*P*-values of the longitudinal comparison confront the characteristics on the day of FET and on the day of the second visit.

In the group of 14 nonpregnant women, the P levels rose, after the FET and the dominant ovarian volume decreased from 15,9 ml ± 1.9 to 12.5 ml ± 1.8 (p 0,034). In the patients who became pregnant after FET (n = 12), no changes were observed in the dominant ovarian volume or vascularization.

## Discussion

The aim of the study was to evaluate whether some of the endometrial or ovarian characteristics before the transfer of frozen and thawed embryo(s) during a spontaneous menstrual cycle predicted the outcome of the FET cycle. At the time of embryo transfer, the only and the most prominent feature in patients who finally conceived was the presence of a triple-line pattern in the endometrium. Otherwise, the patients did not differ in terms of endometrial/subendometrial thickness, volume, or vascularization. Neither the ultrasonographic characteristics in the dominant ovary nor the serum hormonal levels differed between pregnant and nonpregnant subjects at the time of embryo transfer. One week after the transfer, we observed opposite changes in the corpus luteal activity between the successful and unsuccessful cycles.

The presence of a triple-line pattern in the endometrium at FET was strongly associated with the outcome of the cycle. A triple-line pattern reflects endometrial proliferation, and the absence may be a sign of premature secretory changes in the endometrium, suggesting that the time of maximal endometrial receptivity has already passed [12]. Several earlier studies have also observed that a triple-line pattern in the endometrium before embryo transfer is a favorable sign, although in these studies the endometrial pattern was assessed after fresh embryo transfers following controlled ovarian stimulation (COH) [10,13-15]. The only earlier study in which the endometrial pattern was assessed before FET did not find an association between triple-line pattern and cycle outcome [16].

In most of our patients, the triple-line pattern was still visible one week after the FET, about 10 days after the positive LH test. At that time at the midluteal phase, the corpus luteum should be actively secrete progesterone. In addition, all the patients had progesterone supplementation, which is supposed to ensure that in case of corpus luteal deficiency the progesterone concentrations would be high enough. According to earlier findings, a triple-line pattern usually should disappear during the secretory phase under the influence of progesterone secretion[17]. It is possible that, if the second examination in our study had taken place later during the secretory phase, the incidence of triple-line pattern would have been lower. Interestingly, the triple-line pattern was still at the time of implantation more common in patients who eventually conceived.

It was in our interest to evaluate if 3D power Doppler ultrasonography offered us a tool to predict the chances of patients to get pregnant after FET. Since this technique enables the accurate assessment of the volume of the organ and the vasculature supplying the organ, we measured endometrial, subendometrial, and dominant ovarian volumes and their vasculature before FET. The results show however that this technique did not distinguish between successful and unsuccessful cycles and may not be useful for this purpose. This is in accordance with earlier observation after fresh embryo transfer following COH [5,14,18]. There was no difference in endometrial thickness between those who conceived and those who did not, supporting the common opinion that measurement of endometrial thickness cannot be used to predict the likelihood of subsequent conception [19].

There were clear signs of changes in corpus luteal activity with respect to whether the patients conceived or not. In patients who became pregnant, the 17-OHP and E_2 _levels increased, most probably reflecting the rescue of the corpus luteum by hCG secreted from the trophoblastic tissue [20]. On the contrary, in patients who failed, the dominant ovarian volume decreased by the second set of measurements and 17-OHP levels were lower than in pregnant women, reflecting the continuing regression of the corpus luteum without hCG stimulation. The P levels rose in both groups, which probably was due to the exogenous P substitution. It is also very unlikely that the failure of the FET cycle was a consequence of low P levels, since no difference between the groups was observed.

The software in the 3D ultrasonographic equipment provided the determination of echogenity of the tissue by assessing the mean gray value in the region of interest. The use of this modality has been very rare in previous studies [10]. Since the echogenity of the secretory endometrium is usually higher than in the surrounding myometrium, we assessed the echogenity in these two areas subjectively and used software to provide the MG values. A strong correlation between these two methods was observed. Future studies will show whether this index has clinical value.

## Conclusion

The triple-line pattern of the endometrium at the time of FET was the only sign that predisposed pregnancy. Serum steroid levels or the endometrial or ovarian characteristics assessed using 3D power Doppler ultrasonography did not reveal any additional benefit. Nevertheless, more studies with a larger number of women are needed to confirm and extend these findings and establish a more precise cutoff value for endometrial volume, thickness, and vascularization indices.

## List of abbreviations

FET: frozen embryo transfer; IVF: fertilization in vitro; COH: control ovarian hyperstimulation; 17-OHP: 17-hydroxyprogesterone; T: testosterone; hCG: human chorionic gonadotrophin; P: progesterone; E_2_: estradiol; CL: corpus luteum; MG: mean grayness; VI: vascularization index.

## Competing interests

The authors declare that they have no competing interests.

## Authors' contributions

TZ carried out the software analysis of the measurements and wrote the manuscript. IYJ participated in the design of the study, performed the all the 3D-ultrasound examinations, performed the statistical analysis, and participated in writing the manuscript. JST and JF participated in designing the study and writing the manuscript. IYJ was supported by a grant from the Finnish Academy and JST was supported by a grant from the Sigrid Juselius Foundation. All authors read and approved the final manuscript.
